# Supplementary material for: Biodiversity of the Great Barrier Reef—how adequately is it protected?
Source: PeerJ. 2018 May 8;6:e4747. doi: 10.7717/peerj.4747 (PMC5947040; doi:10.7717/peerj.4747)
Supplement: Supplemental Information 1 [file peerj-06-4747-s001.pdf]

## Table S1 – Key threats/ pressures impacting species within the GBR

| Direct or indirect threat | Examples of consequences/ impacts | Implication for species | Example/ reference(s) |
|---|---|---|---|
| **Climate change** | Increasing ocean acidification | • Reduced calcification of corals and other calcifying organisms; reduced species diversity<br>• Habitat destruction, food-web deterioration, disruption of physiological processes, species health and survival<br>• Weakening of carbonate structure | Hannam et al 2007, Veron, et al 2009; Hoegh-Gulberg et al 2007b; Pandolfi et al 2011; Anthony et al 2011; De'ath et al 2013; Dixon et al 2010; Eyre et al 2018 |
| | Rising sea temperatures | • Sea temperature anomalies leading to mass coral bleaching<br>• Range shifts in species<br>• Reduced foraging success for seabirds resulting in increased nesting failures<br>• Impacts on health and reproduction of fishes | Berkelmans et al 2004; Lough 2000; Johnson and Marshall, 2007; Thompson and Dolman 2010; Smithers et al 2003; Donelson et al 2010; Ainsworth et al 2016; Hughes et al 2018 |
| | Changing oceanographic patterns | • Increasing evidence in recent years of intensified flows and increased warming in the East Australian current transporting greater volumes of water southward, carrying larvae and juveniles | Steinberg 2007; Berkelmans et al 2010; Ridgway and Hill 2012; Williams and Crimp 2012 |
| | Rising sand temperatures | • Increasing 'feminization' of Green turtle population already predicted | Fuentes et al 2011; Fuentes et al 2012 |
| | Increased frequency of intense rainfall and increased intensity of severe cyclones | • Increased damage to Reef habitats (e.g. approx. 6% of GBR suffered severe damage during cyclone Yasi; seagrass vulnerable to the effects of reduced light during long periods of exposure to flood plumes); also damage to mangrove habitats (e.g. alter the community structure and productivity)<br>• Increased strandings of species of conservation concern (e.g. dugong and turtle) following seagrass loss due to flooding<br>• Reduced salinity causing bleaching and mortality in corals<br>• Decreased time available for recovery between major disturbances | Done 1992; Lough 2007; Munday et al 2007; Fabricius et al 2008; GBRMPA 2011a; Berkelmans et al 2012 |
| | Rising sea levels | • Even modest sea level rises may have implications for the shape of coastlines and islands<br>• Increased erosion; and inundation leading to significant changes in estuarine habitats<br>• Turtle nesting particularly vulnerable due to greater beach erosion and inundation of nests<br>• Seabird nesting and shorebird roosting sites at risk | Fuentes et al 2010; Church et al 2011 |
| | Ecological consequences due to a loss of a specific level in the food web, a specific species or a specific size class | • Trophic cascades, phase shifts and loss of functional diversity<br>• Reduction in herbivorous species (e.g. herbivorous fish, but also turtles and dugongs) critical to limit macro-algae (seaweed) which otherwise overgrows corals resulting in coral mortality or reduced settlement/ growth of new coral | Marsh et al 2001; Hughes et al 2007; Hoey and Bellwood 2011 |

| | | | |
|---|---|---|---|
| **Declining water quality** | Increased levels of sediments in river plumes | • Increasing sedimentation can smother seagrasses and coral reefs, reduce light penetration in the water column, and impede coral growth and reproduction<br>• Increased turbidity can reduce light available for photosynthesis in seagrasses resulting in "senescence" (shedding of leaves on shoots, thus reducing abundance) and eventual mortality<br>• Increase susceptibility to disease, and suppress subsequent recovery after other disturbances | Devlin et al 2012; McKergrow et al 2005; Wenger et al 2011; Wenger et al 2013; Collier et al 2012; Schroeder et al 2012; Fabricius 2005; Brodie and Pearson 2016 |
| | Increased levels of nutrients in river plumes | • Corals exposed to high levels of nutrients have elevated bleaching susceptibility; nutrient-enriched fine-grained terrestrial silts being particularly detrimental as they are more difficult for corals to shed, causing a barrier to gas exchange and altering microbial communities leading to tissue mortality; dissolved inorganic nutrients can lead to significant physiological changes including decreased calcification<br>• Increased outbreaks of Crown of Thorns seastars (COTs) due to increased phytoplankton (food source for platktonic larval stages of COTs);<br>• Elevated water column nutrients can favour the growth of plankton blooms, macroalgae and epiphytic algae, all of which attenuate light availability to seagrass leaves<br>• Dissolved inorganic nutrients can lead to significant physiological changes such as decreased calcification | Furnas et al 2005; Brodie et al 2005; 2008; 2013; De'ath et al 2012; Waterhouse et al 2013; Jones and Berkelmans 2014; Fabricius et al 2005; Wiedenmann et al 2013; Thompson et al 2014, Brodie and Pearson 2016,; Davis et al 2016 |
| | increased levels of herbicides or pesticides in river plumes | • Chronic herbicide exposure can influence species composition; reduce the efficiency of photosynthesis in seagrasses; in the longer term can cause damage to photosynthesis processes in corals and reduced reproductive output | Lewis et al 2009; Kroon et al 2012; Lewis et al 2012; Smith et al 2012; Turner et al 2013; Shaw et al 2010 |
| | Increased levels of pharmaceuticals and urban pollutants | • Stormwater receives little if any treatment (unlike sewerage) so any chemicals (e.g. fertilizers) enter the GBR without treatment | Harrington, et al 2005; Costanzo et al 2005 |
| | Increased levels of industrial contaminants | • Release of heavy metals, metalloids and non-metalic inorganics<br>• Release of toxic contaminants like mercury, cadmium from old tailings dams; these accumulate up the food chain | Brodie et al 2012; Haynes and Johnson 2000; Angel et al 2010 |
| **Unsustainable coastal development** *(this may occur as part of port activities like dredging or sea dumping, reclamation; or* | Dams, weirs and drainage altering freshwater flows into the GBR | • Construction of artificial barriers or modifications to natural water flows can lead to greater runoff and less water retention on the land.<br>• Modified hydrological processes and alteration of natural coastal processes e.g. changes in the shape and size of river deltas or sand spits | Hyland 2002; Brodie et al 2013; GBRMPA 2013b; Sheaves et al 2014 |
| | Reclamation of marine habitat | • Modifying coastal processes and altering groundwater levels;<br>• Localised impacts on habitats including exposure of acid sulphate soils and changing sediment movement and deposition | Meynecke et al 2007; Sinclair Knight Merz Pty Ltd 2013 |
| | Exposure of acid sulphate soils | • Removal of coastal vegetation can result in the mobilisation of large quantities of iron and aluminium leading to algal blooms<br>• Combination off high acidity waters with heavy metals will affect many species and habitats – often long term impacts that are difficult to reverse | Powell and Martens 2005 |

| | | | |
|---|---|---|---|
| *from urban or industrial expansion)* | Sea dumping | • Burial or smothering of benthic fauna and flora; loss and modification of habitat<br>• Degradation of water quality<br>• Resuspension of sediment deposits by waves and tidal currents leading to burial of seagrass or smothering of coral (previous modelling has seriously underestimated the direction and dispersal; and therefore the potential magnitude and extent of the impacts<br>• Impacts can be exacerbated by the timing and frequency of disposal (e.g. during key spawning times) | Wolanski and Gibbs 1992; Smith et al  2007; Sinclair Knight Merz 2013 |
| | Dredging (both capital and maintenance dredging) | • Loss of species, including benthic organisms; smothering of marine life both at disposal site and in surrounding areas after resuspension<br>• Modifying coastal processes and changing hydrodynamics<br>• Removal or modification of existing habitat like seagrass (which are also huge carbon sinks)<br>• Increase turbidity; changes in species behaviour<br>• Increased underwater noise<br>• Increased sediment affect fish health and survival | Smith et al 2006; Erftemeijer and Lewis 2006; ; Erftemeijer et al 2012; Smith et al 2007; Cagnazzi et al 2013; Pollock et al 2014; Wenger et al 2013; Hess et al 2015 |
| | Increased light pollution and visual disturbance | • Altered light regimes impacting nocturnal orientation of both adult marine turtles and hatchlings<br>• Artificial lighting can cause impacts on seabird hatchlings and some pelagic fish | Kamrowski et al 2012; Santos et al 2010 |
| | Increase in noise | • Noise now considered to be a significant stressor of marine life, particularly marine mammals<br>• Can lead to significant behavioural changes; hearing loss, physical injury and mortality | Slade and Dunlop2014; Ellison et al 2011 |
| | Increase in atmospheric pollution | • Eg. coal dust around port loading facilities, vehicle emissions, ash clouds<br>• Reducing the amount of sunlight reaching the benthos and reduce photosynthetic activity and growth rates | Johnson and Bustin 2006 |
| | Hardening of surfaces | • Increased runoff due to increased roads/hardening | Wong et al 2000 |
| **Shipping impacts** | Noise impacts on marine wildlife | • Direct or indirect fitness costs relation to behavioural and physiological responses to noise pollution | Richardson, et al 1995; UNEP 2012; Francis & Barber 2013 |
| | Vessel strikes on marine wildlife | • Strikes often result in injury or death of marine mammals and turtles | |
| | Ship-sourced pollution | • Waste discharge<br>• Leaching of biocides from ship's anti-foulants | Grech et al 2013; Konstantinou and Albanis 2003 |
| | Introductions of invasive marine pest species | • Consumption of and competition with native coral reef animals<br>• Phase shifts and losses in ecosystem functionality | Worley Parson 2009 |
| | Damage to benthic habitats from ship grounding | • Can significantly impact habitats like coral reefs through either physical damage  (scar from breaking coral) and consequent impacts from anti-foulant paint embedded in coral and paint flakes in sediment | ATSB 2011; GBRMPA 2011b; Turner 2010 |
| | Oil spill/chemical spill from ship grounding or collision | • Apart from the physical smothering of plants and animals, oil toxicity and its chemical reactions means a large spill would have persistent effects on the health, growth, reproduction and survival of many marine species for years, even decades | Aston 2006; Anderson et al 2008 |

| | | | |
|---|---|---|---|
| | Increased turbidity from maintenance dredging and large ships transiting shallow shipping channels | • Fine material from maintenance dredging can be readily transported and resuspended, impacting corals and seagrass many kilometres away from any dumpsite.<br>• Deep-draft ships transiting shallow shipping channels on low tides can be seen from the air as leading to propeller scouring causing resuspension of fine sediments, often onto adjacent areas. | McCook et al. 2015<br><br>Garel et al, 2008 |
| | Damage from repeated ship anchoring eg. close to ports | • Localised chronic effects which can impact biodiversity and cause habitat changes. | GHD P/l 2013; |
| **Unsustainable fishing impacts** | Extraction of top order predators (e.g. sharks) with potential flow-on impacts on habitats and other species in the food web | • Predators play a key role in maintaining healthy ecosystems' reduction in predators can lead to direct and indirect impacts elsewhere in the food chain | Heithaus et al 2008 |
| | Incidental catch of protected species and other species of conservation concern | • Entanglement and/or death of species of high conservation concern | Tobin et al 2010; Courtney et al 2010: GBRMPA and Qld 2013 |
| | Death of non-targeted or discarded (by-catch) species due to size or catch restrictions | • Injure or kill species of conservation concern leading to population declines or compromising the ability of depleted populations to recover | Hall et al 2000; Courtney et al 2007: Pears et al 2012; |
| | Fishing unprotected spawning aggregations of some species | • Declines in fish populations with negative social, economic and ecological consequences | Russell et al 2012; Sadovy and Domeier 2005; Tobin and Currey 2013 |
| | Localised physical damage | • Benthic damage from trawling<br>• Anchor damage and fishing line damage at popular fishing spots eg fringing reefs | Burridge et al 2006; Pitcher et al 2009; Asoh et al 2004 |
| **Disease and pest species** | Outbreaks of COTs | • Major cause of coral mortality when COTS are in outbreak proportions | Furnas et al 2013; Fabricius et al 2010; |
| | Introductions through ballast discharge | • While many such introductions are relatively harmless, some have become aggressive pests | Department of Agriculture, 2013 |
| | Disease outbreaks | • Disease is a clear indicator of stress, especially in species or habitats already under threat from other disturbances (eg. fabropapillomas in turtles already subject to water quality pressures) | Bruno, et al. 2007; Haapkylä et al 2011; Pollock et al 2014; Heron et al 2010 |
| | Toxic blooms | • Can smother seagrass or expose marine species to tumour-producing compounds produced by the cyanobacteria. | Jones 1992; Arthur et al 2006 |
| | Outbreaks of pest species e.g. *Drupella* | • Consumption of benthic organisms with cascading effects including biodiversity loss. Impaired ecosystem functioning, loss of aesthetic value | Cumming 2009 |

| | | | |
|---|---|---|---|
| **Marine debris** | Smother coral or entangle wildlife | • Compromising the ability to capture and digest food, compromising locomotion, including migration and the ability to sense and escape from predators | Reisser et al 2013 |
| | Ingestion causing death or injury | • Decreasing body condition and compromising locomotion and reproduction<br>• Species can absorb persistent, bio-accumulative and toxic substances (eg persistent organic pollutants - POPs), leading to toxicological effects on fish, mammals and molluscs | Andrady 2011; Simmonds 2012; Caron et al 2018 |
| | Discarded fishing gear | • e.g. 'ghost' fishing caused by discarded nets | Laist 1996 |

## REFERENCES CITED IN SUPPLEMENTARY TABLE S1


Ainsworth, T.D., Heron, S.F., Ortiz, J.C., Mumby, P.J., Grech, A., Ogawa, D., Eakin, C.M., Leggat, W. 2016. Climate change disables coral bleaching protection on the Great Barrier Reef. *Science*, 352(6283): 338-342

Andrady, A.L. 2011. Microplastics in the marine environment. *Marine Pollution Bulletin* 62(8): 1596-1605

Anderson, L.E., Melville, F., Jolley, D.F. 2008. An assessment of an oil spill in Gladstone, Australia - impacts on intertidal areas at one-month post-spill. *Marine Pollution Bulletin* 58: 263-271

Angel, B.M., Hales, L.T., Simpson, S.L., Apte, S.C., Chariton, A.A., Shearer, D.A., Jolley, D.F. 2010, Spatial variability of cadmium, copper, manganese, nickel and zinc in the Port Curtis Estuary, Queensland, Australia. *Marine and Freshwater Research* 61(2): 170-183

Anthony, K.R.N., Maynard, J.A., Diaz-Pulido, G., Mumby, P.J., Marshall, P.A., Cao, L., Hoegh-Guldberg, O. 2011. Ocean acidification and warming will lower coral reef resilience. *Global Change Biology* 17: 1798-1808

Arthur, K.E., Limpus, C.J., Roelfsema, C.M., Udy, J.W., Shaw, G.R. 2006. A bloom of Lyngbya majusculain Shoalwater Bay, Queensland, Australia: an important feeding ground for the green turtle (*Chelonia mydas*). *Harmful Algae* 5(3): 251-265

Asoh, A., Yoshikawa, T., Kosaki, R., Marschall, E. 2004. Damage to cauliflower coral by monofilament fishing lines in Hawaii. *Conservation Biology* 18(6): 1645-1650.

Aston, J. 2006. Shipping and oils spills, in The State of the Great Barrier Reef Online, ed. A. Chin, Great Barrier Reef Marine Park Authority, Townsville.

Australian Transport Safety Bureau. 2011. Independent investigation into the grounding of the Chinese registered bulk carrier Shen Neng on Douglas Shoal, Queensland 3 April 2010, ATSB, Canberra

Berkelmans, R., De'ath, G., Kininmonth, S., Skirving, W. J. 2004. A comparison of the 1998 and 2002 coral bleaching events on the Great Barrier Reef: spatial correlation, patterns, and predictions. *Coral Reefs* 23(1): 74-83.

Berkelmans, R., Jones, A.M. and Schaffelke, B. 2012. Salinity thresholds of *Acropora* spp. on the Great Barrier Reef. *Coral Reefs* 31: 1103-1110

Berkelmans, R., Weeks, S.J., Steinberg, C.R. 2010. Upwelling linked to warm summers and bleaching on the Great Barrier Reef. *Limnology and Oceanography* 55(6): 2634-2644

Brodie, J., Binney, J., Fabricius, K., Gordon, I., HoeghGuldberg, O., Hunter, H., O'Reagain, P., Pearson, R., Quirk, M., Thorburn, P., Waterhouse, J., Webster, I., Wilkinson, S. 2008. Scientific consensus statement on water quality in the Great Barrier Reef, Reef Water Quality Protection Plan Secretariat, Brisbane

Brodie, J., Fabricius, K.E., De'ath, G., Okaji, K. 2005. Are increased nutrient inputs responsible for more outbreaks of crown-of-thorns starfish? An appraisal of the evidence. *Marine Pollution Bulletin* 51(1-4): 266-278.

Brodie, J. E., Kroon, F. J., Schaffelke, B., Wolanski, E. C., Lewis, S. E., Devlin, M. J., ... Davis, A. M. 2012. Terrestrial pollutant runoff to the Great Barrier Reef: an update of issues, priorities and management responses. *Marine Pollution Bulletin* 65(4): 81-100

Brodie, J., Waterhouse, J., Schaffelke, B., Kroon, F., Thorburn, P., Rolfe, J., Johnson, J., Fabricius, K., Lewis, S., Devlin, M., Warne, M., McKenzie, L.J. 2013. *2013 Scientific Consensus Statement: Land use impacts on Great Barrier Reef water quality and ecosystem conditions*, Reef Water Quality Protection Plan Secretariat, Brisbane

Brodie, J., Pearson, R. 2016. Ecosystem health of the Great Barrier Reef: time for effective management action based on evidence. *Estuarine, Coastal and Shelf Science* 183: 438-451

Bruno, J.F., Selig, E.R., Casey, K.S., Page, C.A., Willis, B.L., et al. 2007. Thermal stress and coral cover as drivers of coral disease outbreaks. *PloS Biology* 5: e124

Burridge, C.Y., Pitcher, C.R., Hill, B.J., Wassenberg, T.J., Poiner, I.R. 2006. A comparison of demersal communities in an area closed to trawling with those in adjacent areas open to trawling: A study in the Great Barrier Reef Marine Park, Australia. *Fisheries Research* 79(1-2): 64-74

Cagnazzi, D., Parra, G.J., Westley, S., Harrison, P.L. 2013. At the heart of the industrial boom: Australian snubfin dolphins in the Capricorn Coast, Queensland, need urgent conservation action. *PLoS ONE* 8(2): e56729.

Caron, A., Thomas, C., Berry, K., Motti, C., Ariel, E., Brodie, J. 2018. Ingestion of microplastic debris by green sea turtles (*Chelonia mydas*) in the Great Barrier Reef: validation of a sequential extraction protocol. *Marine Pollution Bulletin* 127: 743-751

Church, J.A., White, N.J., Hunter, J.R., McInnes, K.L. 2012. Sea level, in *A marine climate change impacts and adaptation report card for Australia 2012*, ed. E.S. Poloczanska*, et al.*, CSIRO, Canberra, pp. 27-46

Collier, C.J., Waycott, M., McKenzie, L.J. 2012. Light thresholds derived from seagrass loss in the coastal zone of the northern Great Barrier Reef, Australia. *Ecological Indicators* 23: 211-219.

Costanzo, S.D, Murby, J., Bates, J. 2005. Ecosystem response to antibiotics entering the aquatic environment*.* *Marine Pollution Bulletin* 51: 218–223

Courtney, A.J., Haddy, J.A., Campbell, V., Roy, D.P., Tonks, M.L., Gaddes, S.W., Chilcott, K.E., O.Neill, M.F., Brown, I.W., McLennan, M., Jebreen, E.J., van der Geest, C., Rose, C., Kistle, S., Turnbull, C.T., Kyne, P.M., Bennett, M.B., Taylor, J. 2007. *Bycatch weight, composition and preliminary estimates of the impact of bycatch reduction devices in Queensland's trawl fishery*, Department of Primary Industries and Fisheries, Brisbane.

Courtney, A.J., Schemel, B.I., Wallace, R.M., Campbell, M.J., Mayer, D.J. 2010. *Reducing the impact of Queensland's trawl fisheries on protected sea snakes*, Fisheries Research and Development Corporation and Department of Employment, Economic Development and Innovation, Brisbane

Cumming, R. 2009. Population outbreaks and large aggregations of *Drupella* on the Great Barrier Reef, Great Barrier Reef Marine Park Authority, Townsville, Australia.

Davis, A.M., Pearson, R.G., Brodie, J.E., Butler, B. 2016. Review and conceptual models of agricultural impacts and water quality in waterways of the Great Barrier Reef catchment area. *Marine and Freshwater Research* 68 (1): 1-19.

De’ath, G., Fabricius, K.E., Sweatman, H., Puotinen, M. 2012. The 27–year decline of coral cover on the Great Barrier Reef and its causes. *Proceedings of the National Academy of Sciences* 109(44), 17995-17999.

De'ath, G., Fabricius, K., Lough, J. 2013. Yes: Coral calcification rates have decreased in the last twenty-five years! *Marine Geology* 346: 400-402

Department of Agriculture. 2013. Australian Ballast Water Management Requirements (Version 5), Department of Agriculture, Canberra

Devlin, M.J., McKinna, L.I.W., Alvarez-Romero, J.G., Abbott, B., Harkness, P., Brodie, J. 2012. Mapping the pollutants in surface river plume waters in the Great Barrier Reef, Australia. *Marine Pollution Bulletin* 65: 224-235

Dixson, Danielle L., Philip L. Munday, and Geoffrey P. Jones. 2010. Ocean acidification disrupts the innate ability of fish to detect predator olfactory cues. *Ecology Letters* 13: 68-75

Done, T. 1992. Effects of tropical cyclone waves on ecological and geomorphological structures on the Great Barrier Reef. *Continental Shelf Research* 12(7-8): 859-872

Donelson, J.M., Munday, P.L., McCormick, M.I., Pankhurst, N.W., Pankhurst, P.M. 2010. Effects of elevated water temperature and food availability on the reproductive performance of a coral reef fish. *Marine Ecology Progress Series* 401: 233-243.

Ellison, W.T., Southall, B.L., Clark, C.W., Frankel, A.S. 2011. A new context-based approach to assess marine mammal behavioural responses to anthropogenic sounds, *Conservation Biology* 26(1): 21-28

Erftemeijer, P.L., Lewis, R.R.R. 2006. Environmental impacts of dredging on seagrass: a review. *Marine Pollution Bulletin* 52(12): 1553-1572

Erftemeijer, P.L., Riegl, B., Hoeksema, B.W., Todd, P.A. 2012. Environmental impacts of dredging and other sediment disturbances on corals: a review. *Marine Pollution Bulletin* 64(9): 1737-1765.

Eyre, B. D., Cyronak, T., Drupp, P., Heinen De Carlo, E., Sachs, J. P., Andersson, A.J. 2018. Coral reefs will transition to net dissolving before end of century. *Science* 23: 908-911

Fabricius, K. E., De'ath, G. 2001. Biodiversity on the Great Barrier Reef: large-scale patterns and turbidity-related local loss of soft coral taxa. *Oceanographic Processes of Coral Reefs, Physical and Biological Links in the Great Barrier Reef*, pp. 127-144

Fabricius, K.E., De'ath, G., McCook, L.J., Turak, E., Williams, D.M. 2005. Changes in algal, coral and fish assemblages along water quality gradients on the inshore Great Barrier Reef, *Marine Pollution Bulletin* 51(1-4): 384-398

Fabricius, K.E., De'ath, G., Puotinen, M.L., Done, T.J., Cooper, T.F., Burgess, S.C. 2008. Disturbance gradients on inshore and offshore coral reefs caused by a severe tropical cyclone, *Limnology and Oceanography* 53(2): 690-704.

Fabricius, K.E., Okaji, K., De'ath, G. 2010. Three lines of evidence to link outbreaks of the crown-of-thorns seastar *Acanthaster planci* to the release of larval food limitation, *Coral Reefs* 29: 593-605

Francis, C.D., Barber, J.R. 2013. A framework for understanding noise impacts on wildlife: an urgent conservation priority. *Frontiers in Ecology and the Environment* 11: 305–313

Fuentes, M.M.P.B., Limpus, C.J., Hamann, M. 2011. Vulnerability of sea turtle nesting grounds to climate change. *Global Change Biology* 17(1): 140-153

Fuentes, M.M.P.B., Fish, M.R.,Maynard, J.A. 2012. Management strategies to mitigate the impacts of climate change on sea turtle's terrestrial reproductive phase. *Mitigation and Adaptation Strategies for Global Change* 17(1): 51.

Fuentes, M.M.P.B., Limpus, C.J., Hamann, M.,Dawson, J. 2010. Potential impacts of projected sea-level rise on sea turtle rookeries. *Aquatic Conservation: Marine and Freshwater Ecosystems* 20(2): 132-139

Furnas, M., Mitchell, A., Skuza, M., Brodie, J.E. 2005, In the other 90%: Phytoplankton responses to enhanced nutrient availability in the Great Barrier Reef lagoon. *Marine Pollution Bulletin* 51: 253-265

Furnas, M., Brinkman, R., Fabricius, K., Tonin, H., Schaffelke, B. 2013. Chapter 1: Linkages between river runoff, phytoplankton blooms and primary outbreaks of crown-of thorns-seastars in the northern GBR, in Assessment of the relative risk of degraded water quality to ecosystems of the Great Barrier Reef: supporting studies. A report to the Department of Environment and Heritage Protection, Queensland Government, ed. J. Waterhouse, Centre for Tropical Water & Aquatic Ecosystem Research, James Cook University, Townsville

Garel, E., Fernández, L.L., Collins, M. 2008. Sediment resuspension events induced by the wake wash of deep-draft vessels. *Geo-Marine Letters*, 28(4):205-211

GHD Pty Ltd. 2013. Ship anchorage management in the Great Barrier Reef World Heritage Area, Great Barrier Reef Marine Park Authority, Townsville

Great Barrier Reef Marine Park Authority. 2011a. Impacts of tropical cyclone Yasi on the Great Barrier Reef: A report on the findings of a rapid ecological impact assessment, GBRMPA, Townsville

Great Barrier Reef Marine Park Authority. 2011b. Grounding of the Shen Neng 1 on Douglas Shoal, April 2010: impact assessment report, GBRMPA, Townsville

Great Barrier Reef Marine Park Authority. 2013b. *Fitzroy Basin Assessment, Fitzroy Basin Association Natural Resource Management region: Assessment of ecosystem services within the Fitzroy Basin focusing on understanding and improving the health and resilience of the Great Barrier Reef*, GBRMPA, Townsville

Great Barrier Reef Marine Park Authority and Queensland Government. 2013. *Suspicious dugong strandings - Townsville: 2010 to 2013*, Unpublished

Grech, A., Bos, M., Brodie, J., Coles, R., Dale, A., Gilbert, R., Hamann, M., Marsh, H., Neil, K., Pressey, R.L., Rasheed, M.A., Sheaves, M., Smith, A. 2013. Guiding principles for the improved governance of port and shipping impacts in the Great Barrier Reef, *Marine Pollution Bulletin* 75(1-2): 8-20

Haapkylä, J., Unsworth, R.K.F., Flavell, M., Bourne, D.G., Schaffelke, B., Willis, B.L. 2011. Seasonal rainfall and runoff promote coral disease on an inshore reef, *PLoS ONE* 6(2): e16893

Hall, M., Alverson, D. and Metuzals, K. 2000, Bycatch: problems and solutions, *Marine Pollution Bulletin* 41: 204-219

Hamann, M., Limpus, C.J., Read, M.A. 2007. Vulnerability of marine reptiles in the Great Barrier Reef to climate change, in *Climate change and the Great Barrier Reef: a vulnerability assessment*, eds J.E. Johnson and P.A. Marshall, First edn, Great Barrier Reef Marine Park Authority and the Australian Greenhouse Office, Townsville, pp. 465-496

Harrington, L., Fabricius, K., Eaglesham, G., Negri, A.P. 2005. Synergistic effects of diuron and sedimentation on photosynthesis and survival of crustose coralline algae, *Marine Pollution Bulletin* 51: 415-427

Haynes, D. and Johnson, J.E. 2000. Organochlorine, heavy metal and polyaromatic hydrocarbon pollutant concentrations in the Great Barrier Reef (Australia) Environment: a review. *Marine Pollution Bulletin* 41(7-12): 267-278

Heithaus, M.R., Frid, A., Wirsing, A.J., Worm, B. 2008. Predicting ecological consequences of marine top predator declines. *Trends in Ecology & Evolution* 23: 202-210

Heron, S.F., Willis, B.L., Skirving, W.J., Eakin, C.M., Page, C.A., Miller, I.R. 2010. Summer hot snaps and winter conditions: modelling white syndrome outbreaks on Great Barrier Reef corals, *PLoS One* 5(8): e12210

Hess, S., Wenger, A.S., Ainsworth, T.D., Rummer, J.L. 2015. Exposure of clownfish larvae to suspended sediment levels found on the Great Barrier Reef: Impacts on gill structure and microbiome. *Scientific Reports* 5

Hoegh-Guldberg, O., Mumby, P.J., Hooten, A.J., Steneck, R.S., Greenfield, P., Gomez, E., Harvell, C.D., Sale, P.F., Edwards, A.J., Caldeira, K., Knowlton, N., Eakin, C.M., Iglesias-Prieto, R., Muthiga, N., Bradbury, R.H., Dubi, A., Hatziolos, M.E. 2007b, Coral reefs under rapid climate change and ocean acidification *Science* 318: 1737-1742

Hoey, A., Bellwood, D.R. 2011. Suppression of herbivory by macroalgal density: a critical feedback on coral reefs? *Ecology Letters* 14: 267

Hughes, T.P., Rodrigues, M.J., Bellwood, D.R., Ceccarelli, D., Hoegh-Guldberg, O., McCook, L.J., Moltschaniwskyj, N.A., Pratchett, M.S., Steneck, R.S. and Willis, B. 2007, Phase shifts, herbivory, and the resilience of coral reefs to climate change. *Current Biology* 17(4): 360-365

Hughes, T.P., Kerry, J.T., Álvarez-Noriega, M. et al. (43 more authors). 2017. Global warming and recurrent mass bleaching of corals. *Nature* 543 (7645): 373-377

Hyland, S.J. 2002. *An investigation of the impacts of ponded pastures on barramundi and other finfish populations in tropical coastal wetlands: Final project report QO02005*, Fisheries Queensland, Brisbane

Johnson, J.E. and Marshall, P.A. (eds) 2007. *Climate change and the Great Barrier Reef: a vulnerability assessment,* Great Barrier Reef Marine Park Authority and Australian Greenhouse Office, Townsville

Johnson, R. and Bustin, R.M. 2006. Coal dust dispersal around a marine coal terminal (1977-1999), British Colombia: the fate of coal dust in the marine environment. *International Journal of Coal Geology* 68: 57-69

Jones, A.M. and Berkelmans, R. 2014. Flood impacts in Keppel Bay, Southern Great Barrier Reef in the aftermath of cyclonic rainfall. *PLoS ONE* 9(1): e84739

Jones, G.B. 1992. Effect of *trichodesmium* blooms on water quality in the Great Barrier Reef lagoon, in Marine pelagic cyanobacteria: trichodesmium and other diazotrophs, eds E.J. Carpenter, D.G. Capone and J.G. Reuter, Kluwer Academic Press, Dordrecht, pp. 273-287

Kamrowski, R.L., Limpus, C.J., Moloney, J., Hamann, M. 2012. Coastal light pollution and marine turtles: Assessing the magnitude of the problem. *Endangered Species Research* 19: 85-98.

Konstantinou, I., Albanis, T. 2003. Worldwide occurrence and effects of antifouling paint booster biocides in the aquatic environment: A review, *Environment International* 30(2): 235-248

Kroon, F.J., Kuhnert, P.M., Henderson, B.L., Wilkinson, S.N., Kinsey-Henderson, A., Abbott, B., Brodie, J.E., Turner, R.D.R. 2012. River loads of suspended solids, nitrogen, phosphorus and herbicides delivered to the Great Barrier Reef lagoon. *Marine Pollution Bulletin* 65(4-9): 167-181

Laist, D. 1996. Marine debris entanglement and ghost fishing: A cryptic and significant type of bycatch? in Proceedings of the Solving Bycatch Workshop: Considerations for today and tomorrow, Report No 96-03, 25-27 September 1995, Seattle, WA, eds. Alaska Sea Grant, Alaska Sea Grant College Program, Fairbanks, AK, pp.33-39.

Lewis, S.E., Brodie, J.E., Bainbridge, Z.T., Rohde, K.W., Davis, A.M., Masters, B.L., Maughan, M., Devlin, M.J., Mueller, J.F., Schaffelke, B. 2009. Herbicides: A new threat to the Great Barrier Reef. *Environmental Pollution* 157(8-9): 2470- 2484

Lewis, S.E., Schaffelke, B., Shaw, M., Bainbridge, Z.T., Rohde, K.W., Kennedy, K., Davis, A.M., Masters, B.L., Devlin, M.J., Mueller, J.F. 2012. Assessing the additive risks of PSII herbicide exposure to the Great Barrier Reef. *Marine Pollution Bulletin* 65(4-9): 280-291

Lough, J.M. 2007. Climate and climate change on the Great Barrier Reef, in Climate change and the Great Barrier Reef: a vulnerability assessment, eds J.E. Johnson and P.A. Marshall, Great Barrier Reef Marine Park Authority and Australian Greenhouse Office, Townsville, pp. 15-50

Lough, J.M. 2000. 1997–98: Unprecedented thermal stress to coral reefs? *Geophysical Research Letters* 27(23): 3901-3904

Marsh, H., De'ath, G., Gribble, N.A., Lane, B. 2001. *Shark control records hindcast serious decline in dugong numbers off the urban coast of Queensland*, Great Barrier Reef Marine Park Authority, Townsville

Meynecke, J., Lee, S., Duke, N., Warnken, J. 2007. Relationships between estuarine habitats and coastal fisheries in Queensland, Australia, *Bulletin of Marine Science* 80(3): 773-793

Munday, P.L., Jones, G.P., Sheaves, M., Williams, A.J., Goby, G. 2007. Vulnerability of fishes of the Great Barrier Reef to climate change, in Climate change and the Great Barrier Reef: a vulnerability assessment, eds J. Johnson and P.A. Marshall, Great Barrier Reef Marine Park Authority and Australian Greenhouse Office, Townsville, pp. 357-391

McCook, L.L., Schaffelke, B.B., Apte, S.S., Brinkman, R.R., Brodie, J.J., Erftemeijer, P.P., Eyre, B.B., Hoogerwerf, F.F., Irvine, I.I., Jones, R.R., King, B.B. 2015. Synthesis of current knowledge of the biophysical impacts of dredging and disposal on the Great Barrier Reef. Great Barrier Reef Marine Park Authority, Townsville.

McKergrow, L.A., Prosser, I.P., Hughes, A.O., Brodie, J. 2005. Sources of sediment to the Great Barrier Reef World Heritage Area. *Marine Pollution Bulletin* 51: 200

Pandolfi, J.M., Connolly, S.R., Marshall, D.J. and Cohen, A.L. 2011. Projecting coral reef futures under global warming and ocean acidification. *Science* 333(6041): 418-422

Pears, R.J., Morison, A.K., Jebreen, E.J., Dunning, M.C., Pitcher, C.R., Courtney, A.J., Houlden, B., Jacobsen, I.P. 2012. *Ecological risk assessment of the East Coast Otter Trawl Fishery in the Great Barrier Reef Marine Park: Technical report*, Great Barrier Reef Marine Park Authority, Townsville

Pitcher, C.R., Burridge, C.Y., Wassenberg, T.J., Hill, B.J., Poiner, I.R. 2009. A large scale BACI experiment to test the effects of prawn trawling on seabed biota in a closed area of the Great Barrier Reef Marine Park, Australia, Fisheries Research 99(3): 168-183

Pollock, F.J., Lamb, J.B., Field, S.N., Heron, S.F., Schaffelke, B., Shedrawi, G., Bourne, D.G., Willis, B.L. 2014. Sediment and turbidity associated with offshore dredging increase coral disease prevalence on nearby reefs. *PLoS ONE* 9(7): e102498

Powell, B., Martens, M. 2005, A review of acid sulfate soil impacts, actions and policies that impact on water quality in Great Barrier Reef catchments, including a case study on remediation at East Trinity, *Marine Pollution Bulletin* 51: 149- 164

Reisser, J., Shaw, J., Wilcox, C., Hardesty, B.D., Proietti, M., Thums, M.,Pattiaratchi, C. 2013. Marine plastic pollution in waters around Australia: characteristics, concentrations, and pathways. *PLoS ONE* 8(11): e80466

Richardson, W.J., Greene, C.R., Malme, C.I., Thomson, D.H. 1995. Marine mammals and noise, Academic Press, San Diego

Ridgway, K., Hill, K. 2012. East Australian Current, in *A marine climate change impacts and adaptation report card for Australia 2012*, ed. E.S. Poloczanska*, et al.*, CSIRO, Canberra, pp. 47-60

Russell, M.W., Luckhurst, B.E., Lindeman, K.C. 2012. Management of spawning aggregations, in Reef fish spawning aggregations: Biology, research and management, Vol 35, eds Y. Sadovy de Mitcheson and P.L. Colin, Springer Science and Business Media, New York, USA, pp. 371-404

Sadovy, Y., Domeier, M. 2005. Are aggregation-fisheries sustainable? Reef fish fisheries as a case study. *Coral Reefs* 24(2): 254-262

Santos, C.D., Miranda, A.C., Granadeiro, J.P., Lourenco, P.M., Saraiva, S., Palmeirim, J.M. 2010. Effects of artificial illumination on the nocturnal foraging of waders. *Acta Oecologica* 36: 166-172.

Schroeder, T., Devlin, M.J., Brando, V.E., Dekker, A.G., Brodie, J.E., Clementson, L.A. McKinna, L. 2012. Inter-annual variability of wet season freshwater plume extent into the Great Barrier Reef lagoon based on satellite coastal ocean colour observations, *Marine Pollution Bulletin* 65(4-9): 210-223

Shaw, M., Furnas, M.J., Fabricius, K.E., Haynes, D., Carter, S., Eaglesham, G., Muller, J.F. 2010. Monitoring pesticides in the Great Barrier Reef. *Marine Pollution Bulletin* 60(1): 113-122

Simmonds, M.P. 2012. Cetaceans and Marine Debris: The Great Unknown, *Journal of Marine Biology* 2012: 684279

Sinclair Knight Merz Pty Ltd and Asia-Pacific Applied Science Associates 2013, *Improved dredge material management for the Great Barrier Reef Region: Synthesis report*, Great Barrier Reef Marine Park Authority, Townsville

Sheaves, M., Brookes, J., Coles, R., Freckelton, M., Groves, P., Johnston, R., Winberg, P. 2014. Repair and revitalisation of Australia's tropical estuaries and coastal wetlands: opportunities and constraints for the reinstatement of lost function and productivity, *Marine Policy* 47: 23-38

Slade, R.W., Dunlop, R. 2014. Final report: May 2014 survey, monitoring aquatic ambient noise and the associated pressure impacts in Port Curtis and Port Alma. CA130043, Blue Planet Marine

Smith, A., Foster, T., Corcoran, E. and Monkivitch, J. 2007. Dredging and material relocation in sensitive coral environments, in Proceedings of the 18th World Dredging Conference, 1 June, 2007, Florida, eds. Anonymous, Great Barrier Reef Marine Park Authority, Townsville, pp.945-955

Smith, R., Boyd, S.E., Rees, H.L., Dearnaley, M.P., Stevenson, J. 2006. Effects of dredging activity on epifaunal communities - surveys following cessation of dredging. *Estuarine, Coastal and Shelf Science* 70(1-2): 207-223

Smith, R., Middlebrook, R., Turner, R., Huggins, R., Vardy, S., Warne, M. 2012. Large-scale pesticide monitoring across Great Barrier Reef catchments: Paddock to Reef Integrated Monitoring, Modelling and Reporting Program. *Marine Pollution Bulletin* 65(4-9): 117-127

Smithers, B.V., Peck, D.R., Krockenberger, A.K. and Congdon, B.C. 2003. Elevated sea-surface temperature, reduced provisioning and reproductive failure of wedge-tailed shearwaters (*Puffinus pacificus*) in the southern Great Barrier Reef, Australia, *Marine and Freshwater Research* 54(8): 973-97

Steinberg, C. 2007. Impacts of climate change on the physical oceanography of the Great Barrier Reef, in *Climate change and the Great Barrier Reef: a vulnerability assessment*, ed. J.E. Johnson and P.A. Marshall, Great Barrier Reef Marine Park Authority and Australian Greenhouse Office, Townsville, pp. 51-74

Thompson, A.A., Dolman, A.M. 2010. Coral bleaching: one disturbance too many for near-shore reefs of the Great Barrier Reef, *Coral Reefs* 29(3): 637-648

Thompson, A., Schroeder, T., Brando, V., Schaffelke, B. 2014. Coral community responses to declining water quality: Whitsunday Islands, Great Barrier Reef, Australia. Coral Reefs. doi: 10.1007/s00338-014-1201-y

Tobin, A., Currey, L., Simpfendorfer, C. 2013. Informing the vulnerability of species to spawning aggregation fishing using commercial catch data. *Fisheries Research* 143: 47-56.

Tobin, A.J., Simpfendorfer, C.A., Mapleston, A., Currey, L., Harry, A.V., Welch, D.J., Ballagh, A.C., Chin, A., Szczecinski, N., Schlaff, A., White, J.,Moore, B. 2010. *A quantitative ecological risk assessment of sharks and finfish of the Great Barrier Reef World Heritage Area inshore waters: a tool for fisheries and marine park managers. Identifying species at risk and potential mitigation strategies*, Marine and Tropical Sciences Research Facility, Cairns

Turner, A. 2010. Marine pollution from antifouling paint particles. *Marine Pollution Bulletin* 60(2): 159-171

Turner, R., Huggins, R., Wallace, R., Smith, R., Vardy, S. and Warne, M.S.J. 2013. Total suspended solids, nutrient and pesticide loads for rivers that discharge to the Great Barrier Reef: Great Barrier Reef loads monitoring 2010-2011, Water Sciences Technical Report, 81 Volume 2013, Number 1, Department of Science, Information Technology, Innovation and the Arts, Brisbane, Queensland

United Nations Environment Programme. 2012. Scientific synthesis on the impacts of underwater noise on marine and coastal biodiversity and habitats, in Proceedings of the 16th Meeting of the Subsidiary Body on Scientific, Technical and Technological Advice, 30 April - 5 May 2012, Montreal, Canada, eds. UNEP, UNEP, Nairobi, pp.1-93

Veron, J.E.N., Hoegh-Guldberg, O., Lenton, T.M., Lough, J.M., Obura, D.O., Pearce-Kelly, P., Sheppard, C.R., Spalding, M., Stafford-Smith, M.G., Rogers, A.D. 2009. The coral reef crisis: The critical importance of $CO_2$. *Marine Pollution Bulletin* 58(10): 1428-1436

Waterhouse, J., Maynard, J., Brodie, J., Zeh, D., Randall, L., Lewis, S., Petus, C., Devlin, M., da Silva, E., Furnas, M., Schaffelke, B., Fabricius, K., Brando, V., McKensie, L., Collier, C., Warne, M.S.J., Smith, J., Henry, N., Yorkston, H., Tracey, D. 2013. Assessment of the risk of pollutants to ecosystems of the GBR including differential risk between sediments, nutrients and pesticides and among NRM Regions, in Assessment of the relative risk of water quality to ecosystems of the Great Barrier Reef: A report to the Department of the Environment and Heritage Protection, eds J. Brodie, J. Waterhouse, J. Maynard and et al., Centre for Tropical Water & Aquatic Ecosystem Research, James Cook University, Townsville, pp. 18-89

Wenger, A.S., Johansen, J.L., Jones, G.P. 2011. Suspended sediment impairs habitat choice and chemosensory discrimination in two coral reef fishes. *Coral Reefs* 30: 879-887

Wenger, A.S., McCormick, M., McLeod, I.M., Jones, G.P. 2013. Suspended sediment alters predator-prey interactions between two coral reef fish. *Coral Reefs* 32(2): 369- 374

Wiedenmann, J., D'Angelo, C., Smith, E.G., Hunt, A.N., Legiret, F., Postle, A.D., Achterberg, E.P. 2013. Nutrient enrichment can increase the susceptibility of reef corals to bleaching, *Nature Climate Change* 3: 160-164

Williams, K.J., Crimp, S. 2012. *Queensland's biodiversity under climate change: An overview of climate change in Queensland, Climate Adaptation Flagship Working Paper No 12A*, CSIRO Climate Adaptation Flagship, Canberra

Wolanski, E., Gibbs, R. 1992. Resuspension and clearing of dredge spoils after dredging, Cleveland Bay, Australia, Water Environment Research 64(7): 910-914

Wong, T., Breen, P., Lloyd, S. 2000. Water sensitive road design: Design options for improving stormwater quality of road run-off, CRC for Catchment Hydrology, Canberra

Worley Parson. 2009. Benthic macro-invertebrate infauna and introduced marine pest monitoring survey 2009, Report to Far North Queensland Ports Corporation
