# Supplementary material for: Biodiversity of the Great Barrier Reef—how adequately is it protected?
Source: PeerJ. 2018 May 8;6:e4747. doi: 10.7717/peerj.4747 (PMC5947040; doi:10.7717/peerj.4747)
Supplement: Supplemental Information 2 [file peerj-06-4747-s002.docx]

| **Table S2.** List of species known to occur on the Great Barrier Reef that are listed in elevated categories of threat or Data Deficient at global (IUCN) and national (EPBC) scales.  CR = Critically Endangered, EN = Endangered, VU = Vulnerable, DD = Data Deficient. | | | | | | |  |
| --- | --- | --- | --- | --- | --- | --- | --- |
| **Group** | **Species (common name)** | | **IUCN** | | **EPBC** | | |
| Bony Fish | | *Cheilinus undulatus* (Humphead wrasse) | | EN | |  | |
| Bony Fish | | *Bolbometopon muricatum* (Bumphead Parrotfish) | | VU | | X | |
| Bony Fish | | *Cromileptes altivelis* (Humpback Grouper) | | VU | |  | |
| Bony Fish | | *Epinephelus lanceolatus* (Queensland grouper) | | VU | |  | |
| Bony Fish | | *Plectropomus laevis* (Blacksaddled coral grouper) | | VU | |  | |
| Dolphin | | *Stenella longirostris* (Spinner dolphin) | | DD | |  | |
| Dolphin | | *Tursiops aduncus* (Indo-Pacific bottlenose Dolphin) | | DD | |  | |
| Dugong | | *Dugong dugon* (Dugong) | | VU | | X | |
| Hard Coral | | *Acanthastrea bowerbanki* | | VU | |  | |
| Hard Coral | | *Acanthastrea hemprichii* | | VU | |  | |
| Hard Coral | | *Acanthastrea regularis* | | VU | |  | |
| Hard Coral | | *Acropora aculeus* | | VU | |  | |
| Hard Coral | | *Acropora acuminata* | | VU | |  | |
| Hard Coral | | *Acropora anthocercis* | | VU | |  | |
| Hard Coral | | *Acropora aspera* | | VU | |  | |
| Hard Coral | | *Acropora cardenae* | | DD | |  | |
| Hard Coral | | *Acropora caroliniana* | | VU | |  | |
| Hard Coral | | *Acropora dendrum* | | VU | |  | |
| Hard Coral | | *Acropora donei* | | VU | |  | |
| Hard Coral | | *Acropora echinata* | | VU | |  | |
| Hard Coral | | *Acropora elegans* | | VU | |  | |
| Hard Coral | | *Acropora horrida* | | VU | |  | |
| Hard Coral | | *Acropora kirstyae* | | VU | |  | |
| Hard Coral | | *Acropora listeri* | | VU | |  | |
| Hard Coral | | *Acropora microclados* | | VU | |  | |
| Hard Coral | | *Acropora multiacuta* | | VU | |  | |
| Hard Coral | | *Acropora palmerae* | | VU | |  | |
| Hard Coral | | *Acropora paniculata* | | VU | |  | |
| Hard Coral | | *Acropora papillare* | | VU | |  | |
| Hard Coral | | *Acropora polystoma* | | VU | |  | |
| Hard Coral | | *Acropora solitaryensis* | | VU | |  | |
| Hard Coral | | *Acropora speciosa* | | VU | |  | |
| Hard Coral | | *Acropora striata* | | VU | |  | |
| Hard Coral | | *Acropora subglabra* | | VU | |  | |
| Hard Coral | | *Acropora torihalimenda* | | DD | |  | |
| Hard Coral | | *Acropora vaughani* | | VU | |  | |
| Hard Coral | | *Acropora verweyi* | | VU | |  | |
| Hard Coral | | *Acropora willisae* | | VU | |  | |
| Hard Coral | | *Alveopora allingi* | | VU | |  | |
| Hard Coral | | *Alveopora fenestrata* | | VU | |  | |
| Hard Coral | | *Alveopora gigas* | | VU | |  | |
| Hard Coral | | *Alveopora marionensis* | | VU | |  | |
| Hard Coral | | *Alveopora verrilliana* | | VU | |  | |
| Hard Coral | | *Anacropora matthai* | | VU | |  | |
| Hard Coral | | *Anacropora puetogalerae* | | VU | |  | |
| Hard Coral | | *Anacropora reticulata* | | VU | |  | |
| Hard Coral | | *Astreopora cucullata* | | VU | |  | |
| Hard Coral | | *Astreopora incrustans* | | VU | |  | |
| Hard Coral | | *Astreopora moretonensis* | | VU | |  | |
| Hard Coral | | *Australogyra zelli* | | VU | |  | |
| Hard Coral | | *Barabattoia laddi* | | VU | |  | |
| Hard Coral | | *Cantharellus noumeae* | | EN | |  | |
| Hard Coral | | *Catalaphyllia jardinei* | | VU | |  | |
| Hard Coral | | *Caulastrea curvata* | | VU | |  | |
| Hard Coral | | *Caulastrea echinulata* | | VU | |  | |
| Hard Coral | | *Cyphastrea agassizi* | | VU | |  | |
| Hard Coral | | *Cyphastreea ocellina* | | VU | |  | |
| Hard Coral | | *Euphyllia ancora* | | VU | |  | |
| Hard Coral | | *Euphyllia cristata* | | VU | |  | |
| Hard Coral | | *Favia rosaria* | | VU | |  | |
| Hard Coral | | *Fungia curvata* | | VU | |  | |
| Hard Coral | | *Galaxea acrhelia* | | VU | |  | |
| Hard Coral | | *Galaxea astreata* | | VU | |  | |
| Hard Coral | | *Heliopofungia actiniformis* | | VU | |  | |
| Hard Coral | | *Heliopora coerulea* | | VU | |  | |
| Hard Coral | | *Isopora brueggemanni* | | VU | |  | |
| Hard Coral | | *Isopora crateriformis* | | VU | |  | |
| Hard Coral | | *Isopora cuneata* | | VU | |  | |
| Hard Coral | | *Leptoria irregularis* | | VU | |  | |
| Hard Coral | | *Leptoseris incrustans* | | VU | |  | |
| Hard Coral | | *Leptoseris yabei* | | VU | |  | |
| Hard Coral | | *Lobophyllia diminuta* | | VU | |  | |
| Hard Coral | | *Lobophyllia flabelliformis* | | VU | |  | |
| Hard Coral | | *Millepora foveolata* | | VU | |  | |
| Hard Coral | | *Montastrea salebrosa* | | VU | |  | |
| Hard Coral | | *Montipora angulata* | | VU | |  | |
| Hard Coral | | *Montipora australiensis* | | VU | |  | |
| Hard Coral | | *Montipora caliculata* | | VU | |  | |
| Hard Coral | | *Montipora capricornis* | | VU | |  | |
| Hard Coral | | *Montipora corbettensis* | | VU | |  | |
| Hard Coral | | *Montipora crassituberculata* | | VU | |  | |
| Hard Coral | | *Montipora turtlensis* | | VU | |  | |
| Hard Coral | | *Moseleya latistellata* | | VU | |  | |
| Hard Coral | | *Pavona cactus* | | VU | |  | |
| Hard Coral | | *Pavona decussata* | | VU | |  | |
| Hard Coral | | *Pavona venosa* | | VU | |  | |
| Hard Coral | | *Pectinia alicornis* | | VU | |  | |
| Hard Coral | | *Pectinia lactuca* | | VU | |  | |
| Hard Coral | | *Physogyra lichtensteini* | | VU | |  | |
| Hard Coral | | *Pocillopora danae* | | VU | |  | |
| Hard Coral | | *Porites evermannai* | | DD | |  | |
| Hard Coral | | *Porites nigrescens* | | VU | |  | |
| Hard Coral | | *Turbinaria bifrons* | | VU | |  | |
| Hard Coral | | *Turbinaria heronensis* | | VU | |  | |
| Hard Coral | | *Turbinaria mesenterina* | | VU | |  | |
| Hard Coral | | *Turbinaria patula* | | VU | |  | |
| Hard Coral | | *Turbinaria peltata* | | VU | |  | |
| Hard Coral | | *Turbinaria reniformis* | | VU | |  | |
| Hard Coral | | *Turbinaria stellulata* | | VU | |  | |
| Mollusc | | *Hipopus hippopus* | | DD | |  | |
| Mollusc | | *Tridacna derasa* (Southern Giant Clam) | | VU | |  | |
| Mollusc | | *Tridacna gigas*(Giant Clam) | | VU | |  | |
| Ray | | *Anoxypristis cuspidata* (Narrow sawfish) | | EN | |  | |
| Ray | | *Dasyatis fluviorum* (Estuary stingray) | | VU | |  | |
| Ray | | *Dasyatis microps* (Smalleye Stingray) | | DD | |  | |
| Ray | | *Dipturus melanospilus* (Blacktip Skate) | | DD | |  | |
| Ray | | *Dipturus queenslandicus* (Queensland Deepwater Skate) | | DD | |  | |
| Ray | | *Insentiraja laxipella* (Eastern Looseskin Skate) | | DD | |  | |
| Ray | | *Manta alfredi* (Reef Manta) | | VU | | X | |
| Ray | | *Manta birostris* (Giant Manta) | | VU | | X | |
| Ray | | *Mobula thurstoni* (Bentfin Devilray) | | VU | |  | |
| Ray | | *Myliobatus hamlyni* (Purple eagle ray) | | EN | |  | |
| Ray | | *Neotrygon kuhlii* (Blue-spotted Maskray) | | DD | |  | |
| Ray | | *Notoraja ochroderma* (Pale Skate) | | DD | |  | |
| Ray | | *Pristis clavata* (Dwarf Sawfish) | | EN | | X | |
| Ray | | *Pristis pristis* (Indo-West Pacific Largetooth Sawfish) | | CR | | X | |
| Ray | | *Pristis zijsron (*Green Sawfish) | | CR | | X | |
| Ray | | *Rhinpotera neglecta* (Australian Cownose ray) | | DD | |  | |
| Ray | | *Torpedo macneilli* (Short-tail torpedo ray) | | DD | |  | |
| Ray | | *Torpedo tokionis* (Long-tail torpedo ray) | | DD | |  | |
| Sea cucumber | | *Actinopyga echinites* (Deepwater redfish) | | VU | |  | |
| Sea cucumber | | *Actinopyga mauritiana* (Surf redfish) | | VU | |  | |
| Sea cucumber | | *Actinopyga miliaris* (Hairy blackfish) | | VU | |  | |
| Sea cucumber | | *Holothuria fuscogilva* (White teatfish) | | VU | |  | |
| Sea cucumber | | *Holothuria lessoni* (Golden sandfish) | | EN | |  | |
| Sea cucumber | | *Holothuria nobilis* (Black teatfish) | | EN | |  | |
| Sea cucumber | | *Holothuria scabra* (Sandfish) | | EN | |  | |
| Sea cucumber | | *Holothuria whitmaei* (Black teatfish) | | EN | |  | |
| Sea cucumber | | *Stichopus herrmanni* (Curryfish) | | VU | |  | |
| Sea cucumber | | *Thelenota ananas* (Prickly redfish) | | EN | |  | |
| Shark | | *Atelomycterus marnkalha* (Eastern Banded Catshark) | | DD | |  | |
| Shark | | *Brachaelurus colcloughi* (Colclough’s Shark) | | VU | |  | |
| Shark | | *Carcharhinus longimanus* (Oceanic Whitetip Shark) | | CR | |  | |
| Shark | | *Carcharias taurus* (grey nurse shark, east coast population) | | CR | | X | |
| Shark | | *Centrophorus granulosus* (Gulper Shark) | | VU | |  | |
| Shark | | *Cephaloscyllium signourum* (Speckled Swellshark) | | DD | |  | |
| Shark | | *Cephaloscyllium zebrum* (Narrowbar Swellshark) | | DD | |  | |
| Shark | | *Chimaera obscura* (Shortspine Chimaera) | | DD | |  | |
| Shark | | *Figaro striatus* (Northern Sawtail Shark) | | DD | |  | |
| Shark | | *Galeus gracilis* (Slender Sawtail shark) | | DD | |  | |
| Shark | | *Glyphis glyphis* (Speartooth Shark) | | EN | | X | |
| Shark | | *Hexanchus nakamurai* (Bigeye Sixgill Shark) | | DD | |  | |
| Shark | | *Isurus oxyrinchus* (Shortfin Mako) | | VU | |  | |
| Shark | | *Mustelus walker* (Whitespotted Gummyshark) | | DD | |  | |
| Shark | | *Parmaturus bigus* (Short-tail Catshark) | | DD | |  | |
| Shark | | *Sphyrna lewini* (Scalloped Hammerhead Shark) | | EN | |  | |
| Shark | | *Sphyrna mokarran* (Great Hammerhead Shark) | | EN | |  | |
| Shark | | *Squalus montalbani* (Greeneye Spurdog) | | VU | |  | |
| Shark | | *Squalus notocaudatus* (Bartail Spurdog) | | DD | |  | |
| Shark | | *Squatina albipunctata* (Eastern Angelshark) | | VU | |  | |
| Shark | | *Carcharodon carcharias* (Great White Shark) | | EN | | X | |
| Shark | | *Rhincodon typus* (Whale Shark) | | VU | | X | |
| Turtle | | *Caretta caretta* (Loggerhead) | | EN | | X | |
| Turtle | | *Chelonia mydas* (Green Turtle) | | EN | | X | |
| Turtle | | *Dermochelys coriacea* (Leatherback) | | VU | | X | |
| Turtle | | *Eretmochelys imbricata* (Hawksbill turtle) | | CR | | X | |
| Turtle | | *Lepidochelys olivacea* (Olive Ridley ) | | VU | | X | |
| Turtle | | *Natator depressus* (Flatback turtle) | | DD | | X | |
| Whale | | *Balaenoptera borealis* (Sei Whale) | | EN | | X | |
| Whale | | *Balaenoptera edeni* (Brydes Whale) | | DD | |  | |
| Whale | | *Balaenoptera musculus* (Blue Whale) | | EN | | X | |
| Whale | | *Balaenoptera physalus* (Fin Whale) | | EN | | X | |
| Whale | | *Feresa attenuata* (Pygmy Killer Whale) | | DD | | X | |
| Whale | | *Globicephala macrorhynchus* (short-finned pilot whale) | | DD | |  | |
| Whale | | *Globicephala melas* (Long-finned pilot whale) | | DD | |  | |
| Whale | | *Indopacetus pacificus* (Tropical bottle nosed whale) | | DD | | X | |
| Whale | | *Kogia breviceps* (Pygmy sperm whale) | | DD | |  | |
| Whale | | *Kogia sima* (Dwarf Sperm Whale) | | DD | |  | |
| Whale | | *Orcinus orca* (Killer Whale) | | DD | |  | |
| Whale | | *Physeter macrocephalus* (Sperm whale) | | VU | | X | |
| Whale | | *Pseudorca crassidens* (False Killer Whale) | | DD | |  | |
